# Impaired c-Fos and Polo-Like Kinase 2 Induction in the Limbic System of Fear-conditioned α-Synuclein Transgenic Mice

**DOI:** 10.1371/journal.pone.0050245

**Published:** 2012-11-27

**Authors:** Heinrich Schell, Cindy Boden, André Maia Chagas, Philipp J. Kahle

**Affiliations:** 1 Laboratory of Functional Neurogenetics, Department of Neurodegeneration, Hertie Institute for Clinical Brain Research, University of Tübingen, Tübingen, Germany; 2 German Center for Neurodegenerative Diseases, University of Tübingen, Tübingen, Germany; 3 Graduate School of Cellular and Molecular Neuroscience, University of Tübingen, Tübingen, Germany; National Institute of Health, United States of America

## Abstract

α-Synuclein (αSYN) is genetically and neuropathologically linked to a spectrum of neurodegenerative diseases including Parkinson’s disease, dementia with Lewy bodies, and related disorders. Cognitive impairment is recapitulated in several αSYN transgenic mouse lines. However, the mechanisms of dysfunction in affected neurons are largely unknown. Here we measured neuronal activity induced gene products in the limbic system of αSYN transgenic mice upon fear conditioning (FC). Induction of the synaptic plasticity marker c-Fos was significantly reduced in the amygdala and hippocampus of (Thy1)-h[A30P]αSYN transgenic mice in an age-dependent manner. Similarly, the neuronal activity inducible polo-like kinase 2 (Plk2) that can phosphorylate αSYN at the pathological site serine-129 was up-regulated in both brain regions upon FC. Plk2 inductions were also significantly impaired in aged (Thy1)-h[A30P]αSYN transgenic mice, both in the amygdala and hippocampus. Plk2 inductions in the amygdala after FC were paralleled by a small but significant increase in the number of neuronal cell bodies immunopositive for serine-129 phosphorylated αSYN in young but not aged (Thy1)-h[A30P]αSYN transgenic mice. In addition, we observed in the aged hippocampus a distinct type of apparently unmodified transgenic αSYN profiles resembling synaptic accumulations of αSYN. Thus, the cognitive decline observed in aged αSYN transgenic mice might be due to impairment of neurotransmission and synaptic plasticity in the limbic system by distinct αSYN species.

## Introduction

α-Synuclein (αSYN) fibrils are the major building blocks of Lewy bodies (LBs) and Lewy neurites, which comprise the neuropathological hallmarks of Parkinson’s disease (PD) and related disorders [1]. The amygdala is one predilection site of Lewy pathology in dementia with LBs (DLB) and also in Alzheimer’s disease [2], [3]. In addition, there are synaptic αSYN accumulations in the hippocampal formation of human α-synucleinopathy patients and α-synuclein transgenic mouse models [4], [5]. Moreover, point mutations [6], [7], [8] as well as genomic multiplications of the gene encoding αSYN cause not only PD motor symptoms, but also dementia in a gene dose dependent manner [9]. Several αSYN transgenic mouse models have been developed in the past [10], and cognitive impairments in such mouse models are emerging [4], [11], [12]. Cognitive deficits have been correlated with αSYN neuropathology in the amygdala and hippocampus. However, it remains largely unknown if and how α-synucleinpathy affects neurotransmission and synaptic plasticity *in vivo*.

Here we have investigated neuronal plasticity in the limbic system (amygdala and hippocampus) upon fear conditioning (FC) in a transgenic mouse model expressing human A30P mutant [6] αSYN under control of a CNS neuron predominant Thy1 promotor [13], [14], [15]. These animals show age-dependent decline of emotional learning concomitant with αSYN alterations in the amygdala [11], [16]. Mice were FC trained and sacrificed within an hour for quantitative immunohistological examination of the neuronal plasticity marker c-Fos [17]. This method was also used to measure cognitive impairment in transgenic mouse models of Alzheimer’s disease [18], [19]. In addition, we examined the neuronal activity responsive gene product, polo-like kinase 2 (Plk2) [20], [21] since this enzyme is an important enzyme phosphorylating αSYN at the pathological site serine-129 (pSer129) [22], [23]. As the (Thy1)-h[A30P]αSYN mice aged and became impaired in FC behavior, they showed significantly reduced c-Fos and Plk2 induction compared to wild-type control mice, both in the amygdala and in the hippocampus. We attempt to correlate the age-dependent impairments in synaptic plasticity and cognitive behavior with the development of various forms of αSYN and pSer129 neuropathologies within the limbic system of (Thy1)-h[A30P]αSYN mice, including synaptic accumulations of apparently “normal” transgenic αSYN in the hippocampus. Distinct αSYN species may cause age-dependent impairments in synaptic plasticity during FC learning paradigms via multiple mechanisms, which might be relevant for the development of dementia in human patients.

## Materials and Methods

### Ethics Agreement

The behavioral tests and brain dissections were in compliance with the authorization N10/08 licensed by the regional board (Regierungspräsidium) Tübingen and were performed according to the German law, Guide for the Care and Use of Laboratory Animals.

### Fear Conditioning

All behavioral tests were done with male mice. FC was conducted as described before [11], [16]. Briefly animals were habituated one week before the FC training. In this period only the experimenter took care of the mice. Two to three days before the FC animals were involved in 1–2 handling sessions per day so that animals did not display any indication of anxiety before FC in a system from TSE (Bad Homburg, Germany). During the 6 min training session animals were exposed to either none or two 0.6 mA scrambled foot shocks for 1s, which was announced by a 20 s light/tone cue immediately prior to shock. Within 40–60 min after the training, half of the animals were sacrificed by cervical dislocation and dissected brains were fixed in 4% paraformaldehyde in phosphate buffer (pH 7.4). The other half of the group was exposed 24h later for 3 min to the previous FC context. In this time exploratory behavior of each animal was recorded. After another 6 h animals were placed in a changed context, in which the clear Perspex walls of the test cage were replaced with black walls. A Perspex plate covered with woodchips was placed over the foot shock grids. In this new context the mice were exposed to the light/tone cue used in the training for 3 min, during which exploration was recorded. For both the context test as well as the cued test automated recordings, the explored area was represented as a virtual field consisting of 256 elements. The percentage of visited area out of the whole field was determined.

### Histological Staining Procedures

Animals were sacrificed within 40–60 min after training, brains dissected and subjected to histological analyses. Fixed brains were embedded in paraffin and 4 µm thick coronary serial sections were cut on a microtome and directly transferred onto SuperFrost Plus coverslips (Fisher Scientific, Schwerte, Germany). Serial sections containing basal amygdala (BA), lateral amygdala (LA) and the central amygdaloid nucleus (CE) as well as the hippocampus were taken for histological staining.

For immunostainings, primary antibodies were rabbit polyclonal antibody against c-Fos (sc-52, Santa Cruz Biotechnology, Santa Cruz, CA, USA; 1∶100) and rabbit polyclonal antibody against Plk2 (BL1695, Bethyl Laboratories, Montgomery, TX, USA; 1∶200), rat monoclonal antibody against human αSYN (15G7 [13]; 1∶5) or rabbit monoclonal anti-pSer129 (clone EP1536Y, Epitomics, Burlingame, CA, USA; 1∶500). After overnight incubation at 60°C and treatment with xylene, rehydration was performed over a descending ethanol series: 100%, 95%, 75%, Tris-buffered saline, pH 7.4 (TBS). Peroxidase activity was eliminated by a 30 min treatment with 1% hydrogen peroxide followed by 3 washing steps with TBS. Antigen retrieval was done by a 30 min exposure of the sections to citrate buffer (pH 6.0) at 90°C, followed by a cooling step on ice. Blocking was done in 5% normal goat serum in TBS for 30 min at room temperature. Incubation with primary antibodies was done in TBS with 2% normal goat serum at 4°C overnight. After 3 washing steps with TBS sections were incubated for 1 h at room temperature with biotinylated goat anti-rabbit antibody followed by the avidin-biotin-complex (Vectastain ABC kit). Vector SG-Blue and 5 min incubation with nuclear fast red were used for counterstaining. After dehydration (ethanol: 75%, 95%, 100%, xylene) sections were mounted with Pertex. Slides stained with Thioflavin-S (ThS) were incubated after the antigen retrieval step for 15 min in 0.1% ThS aqueous solution under light protection. After three 5 min washing steps under light protection in 70% ethanol, slides were mounted with Moviol (DABCO). Gallyas silver staining was performed after deparaffinization of the tissue. Slides were incubated for 5 min in 5% periodic acid, washed two times for 5 min in water, followed by a 1 min incubation step in silver iodide under constant gentle agitation. Then slides were incubated for 10 min in 0.5% acetic acid and developed. After developing tissue was incubated for 3 min in 0.5% acetic acid, followed by a 5 min incubation step in water and another 5 min incubation step in 0.1% gold chloride. Afterwards slides were rinsed in water, incubated for 5 min in 1% sodium thiosulfate and rinsed again in water. Counterstaining with nuclear fast red was performed for 5 min, followed by the dehydration of the tissue (ethanol: 75%, 95%, 100%, xylene) and mounting with Pertex.

Photomicrogaphs were taken with an Axioplan 2 Imaging microscope (Carl Zeiss, Jena, Germany) and processed with the AxioVision 4.3 imaging software. For stereology the stereo investigator software (MBF Bioscience, Williston, VT, USA) with the option “area fraction fractionator” was used. LA and BA amygdala regions were compiled together and were quantified as basolateral amygdala (BLA). Stainings and stereology were performed double-blindly by two experimenters.

## Results

**Figure 1:**
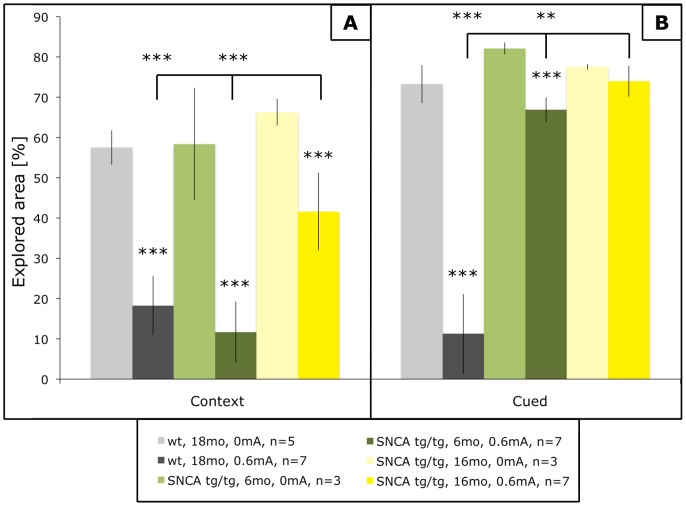
Impaired FC in aging (Thy1)-h[A30P]αSYN mice. The indicated groups of mice were trained and after 24 h assessed for context learning (A) and after another 6 h for cued learning (B). Compared to non-shocked control mice (light bars), age-matched old trained wild-type (wt) mice (gray bars) showed highly significant reductions of exploratory behavior in response to both context and cues. Young (Thy1)-h[A30P]αSYN transgenic (tg) mice (green bars) showed almost normal FC for context and showed significantly reduced exploratory behavior in cued FC when compared to old transgenic mice (yellow bars). The old transgenic mice showed much reduced context FC and no cued FC at all. ***p<0.0001; **p<0.01.

The (Thy1)-h[A30P]αSYN transgenic mice were shown before to develop age-dependent deterioration in FC behavior concomitant with αSYN aberrations in the amygdala [11], [16]. FC acquisition (shock-trained freezing) was no different among the animal groups (results not shown). Within 40–60 min after training, mice were sacrificed, brains dissected and subjected to histological analyses. Parallel animals were left for context and cue recalls, confirming in this set of experiments the age-dependent impairments in both FC paradigms (Fig. 1), particularly in the cued-test that depends on amygdala function but also the context-test that involves the hippocampus [24], [25].

**Figure 2:**
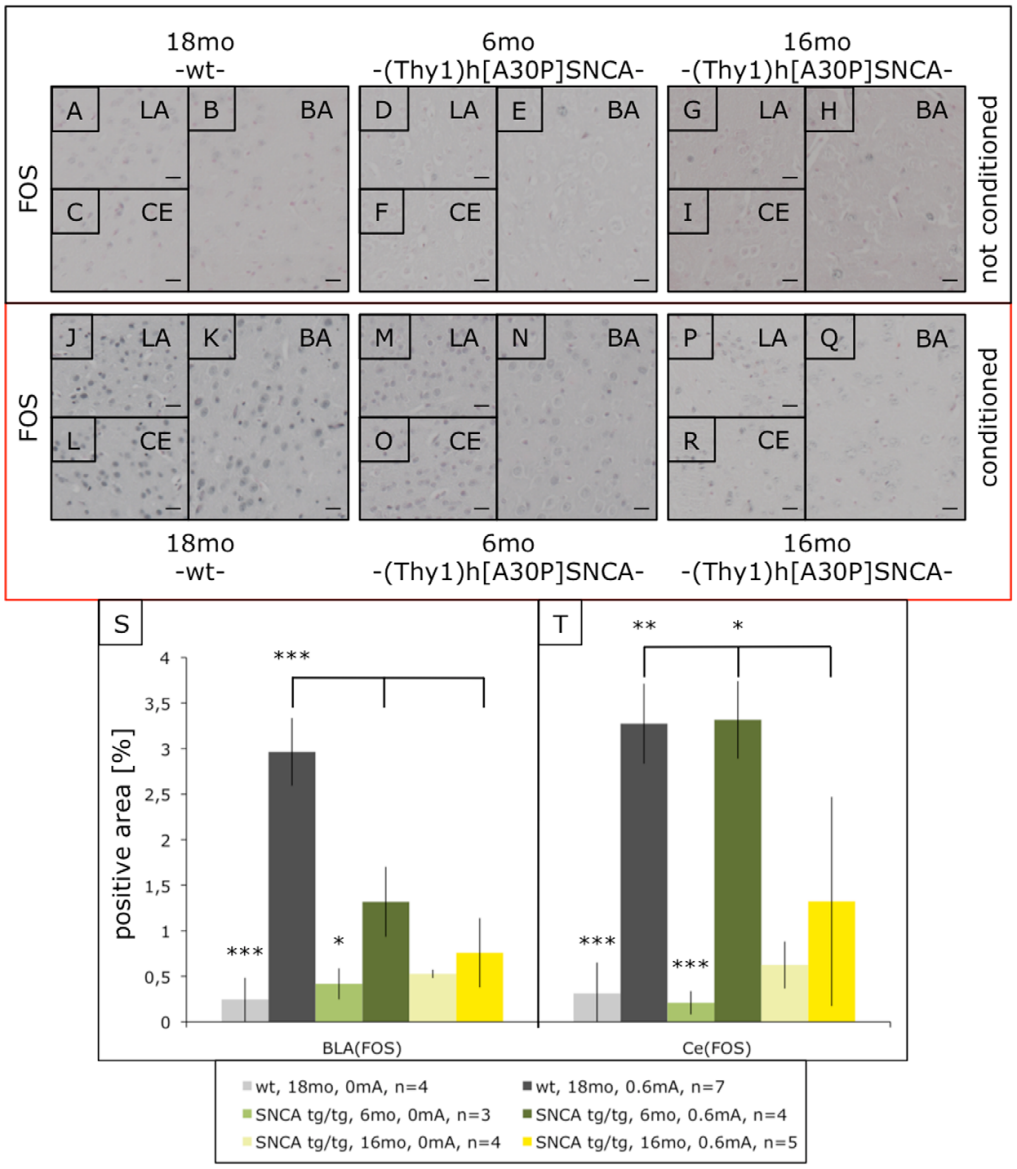
c-Fos immunostaining in the amygdala of fear-conditioned (Thy1)-h[A30P]αSYN mice. Parallel mice as in Figure 1 were sacrificed within 40–60 min after FC training, brains dissected and amygdala sections stained with an antibody against c-Fos. Hardly any c-Fos immunoreactivity was observed in non-shocked mice under these conditions (A–I), whereas shock training induced c-Fos signals even in old non-transgenic mice (J–L). In contrast, young transgenic mice showed reduced c-Fos induction (M–O), which in the case of old (Thy1)-h[A30P]αSYN mice was so much impaired throughout the amygdala (P–R) that it failed to reach significance (S,T). ***p<0.0001; **p<0.001; *p<0.02. Size bars correspond to 20 µm. LA, lateral amygdala, BA, basal amygdala, CE, central nucleus.

First we measured by quantitative immunostaining the immediate early gene product c-Fos known to be induced during FC synaptic plasticity [17]. As expected, c-Fos immunoreactivity was massively increased in the amygdala after FC of C57Bl/6 wild-type mice even aged 18mo (Fig. 2). Likewise, c-Fos was up-regulated in 6mo young (Thy1)-h[A30P]αSYN mice, but less significantly compared even to old wild-type mice, whereas 16mo old transgenic mice showed no significant c-Fos induction in the amygdala (Fig. 2), consistent with the inability to perform in the cued fear conditioning test (Fig. 1).

**Figure 3:**
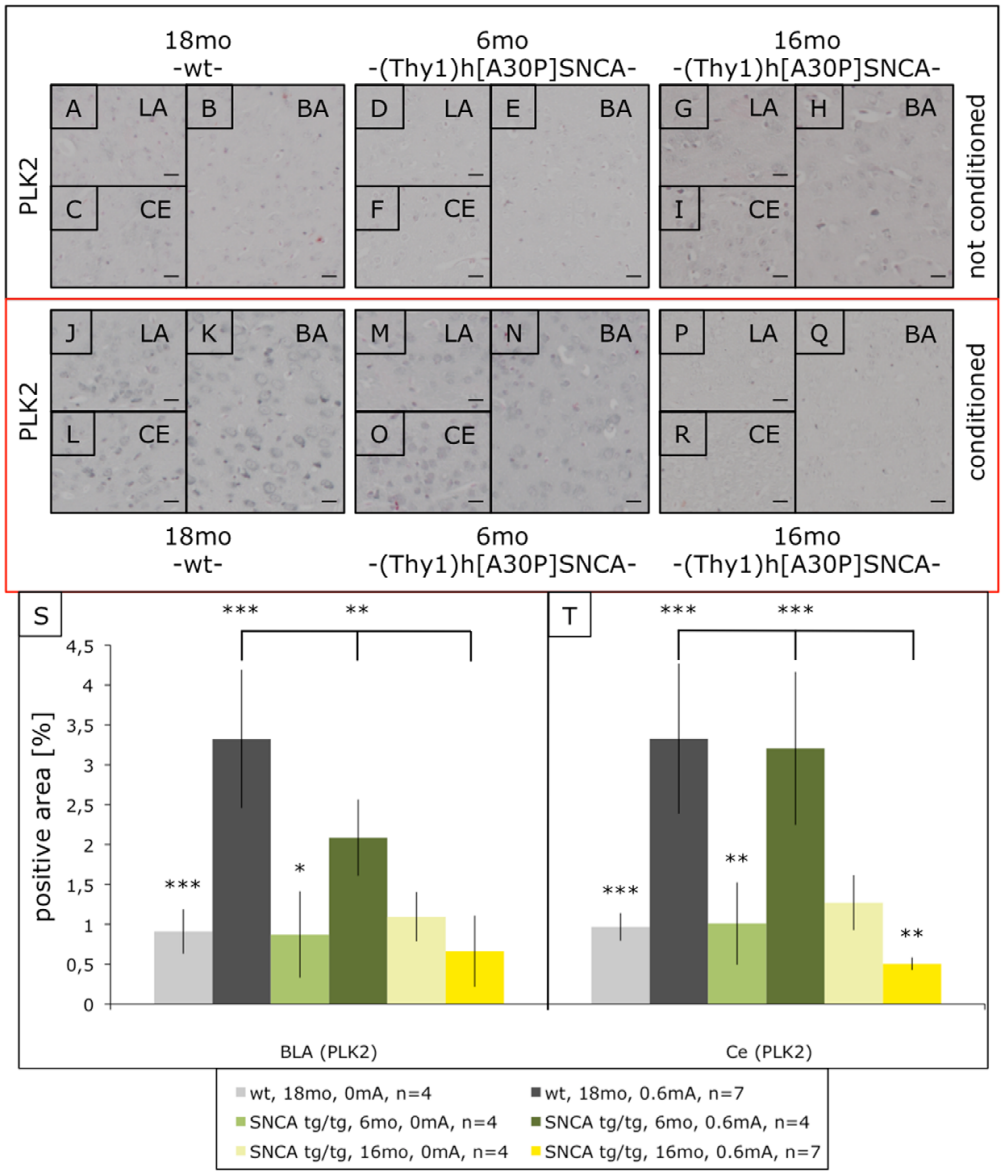
Plk2 immunostaining in the amygdala of fear-conditioned (Thy1)-h[A30P]αSYN mice. Mice were processed as above and amygdala sections stained with an antibody against Plk2. Compared to non-shocked mice (A–I), FC induced Plk2 signals even in old non-transgenic mice (J–L). In contrast, young transgenic mice showed reduced Plk2 induction (M–O), which in the case of old (Thy1)-h[A30P]αSYN mice was almost absent (P–R). Size bars correspond to 20 µm. Quantifications show statistical significances (S,T). ***p<0.001; **p<0.01; *p<0.02. LA, lateral amygdala, BA, basal amygdala, CE, central nucleus.

Next we analyzed Plk2 because it is a kinase effectively phosphorylating αSYN at the pathological site serine-129 [22], [23] and acts as a suppressor in simple animal models of αSYN toxicity [26]. Moreover, Plk2/Snk is induced by neuronal activity [20], [21]. We found for the first time that synaptic plasticity during FC up-regulated Plk2 in the amygdala of wild-type mice even as old as 18mo (Fig. 3). As for c-Fos, already 6mo young (Thy1)-h[A30P]αSYN mice showed somewhat reduced Plk2 induction, and 16mo old transgenic mice were completely deficient in inducing Plk2 in the amygdala after FC (Fig. 3). In parallel we observed a small but significant increase in pSer129-positive neurons upon FC in young (Thy1)-h[A30P]αSYN mice, which was absent in the old animals (Fig. S1).

**Figure 4:**
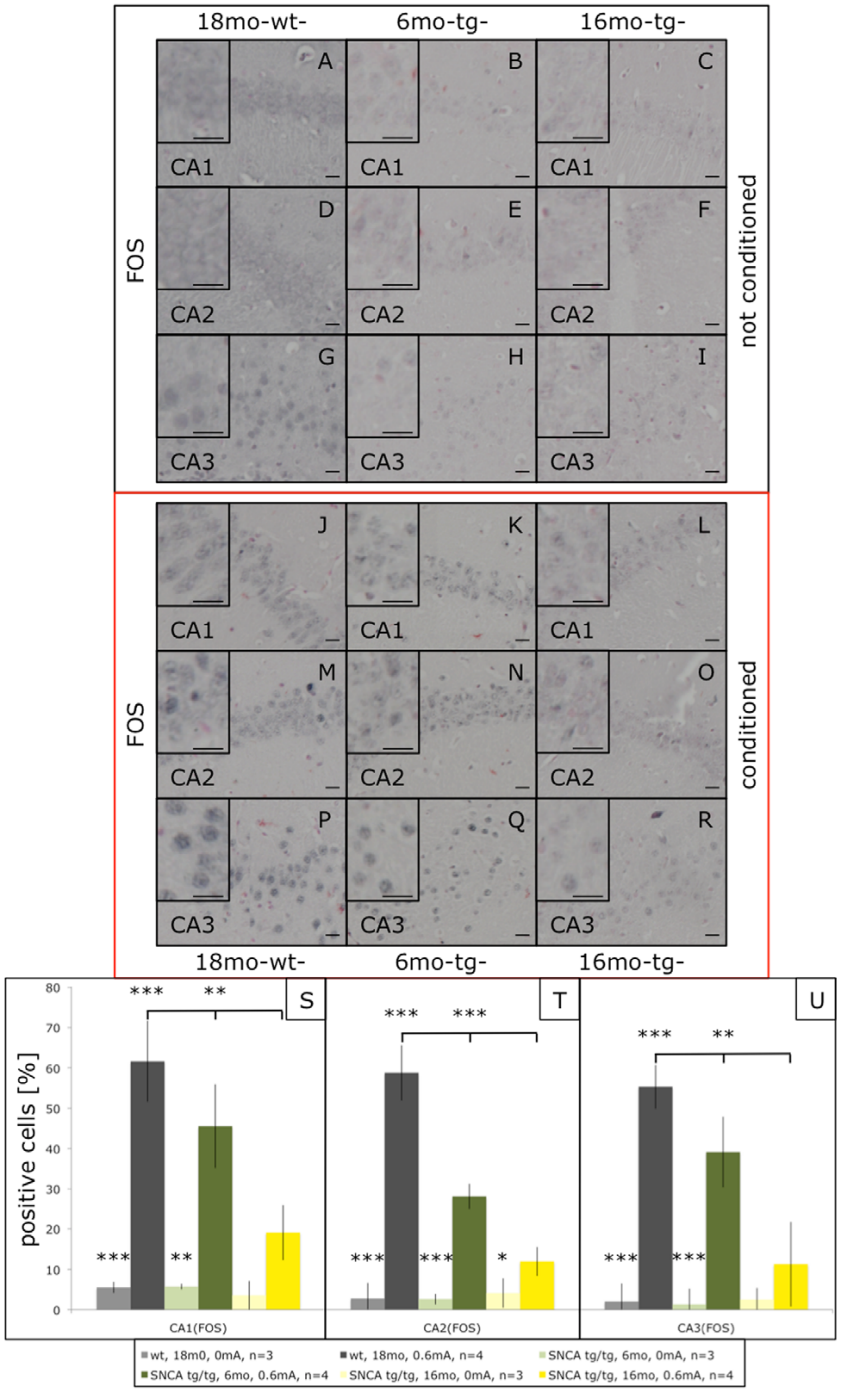
c-Fos immunostaining in the hippocampus of fear-conditioned (Thy1)-h[A30P]αSYN mice. Mice were processed as described above and hippocampal sections stained with an antibody against c-Fos. Compared to non-shocked mice (A–I), FC induced c-Fos signals in the hippocampal regions CA1, CA2, and CA3 of old non-transgenic mice (J–P) and young (Thy1)-h[A30P]αSYN mice (K–Q). This FC dependent up-regulation of c-Fos was significant for the old wt group (grey bars in S, T, U; ***p<0.003) and the young transgenic mice (green bars in S, T, U; ***p<0.008; **p<0.0016). In contrast, c-Fos up-regulation was reduced in old transgenic mice (L–R) and showed only a small significance in CA2 (yellow bars in T; *p<0.0357), but failed to show any significance in the CA1 and CA3 region of the conditioned old transgenic group (yellow bars in S and U). Size bars correspond to 20 µm.

**Figure 5:**
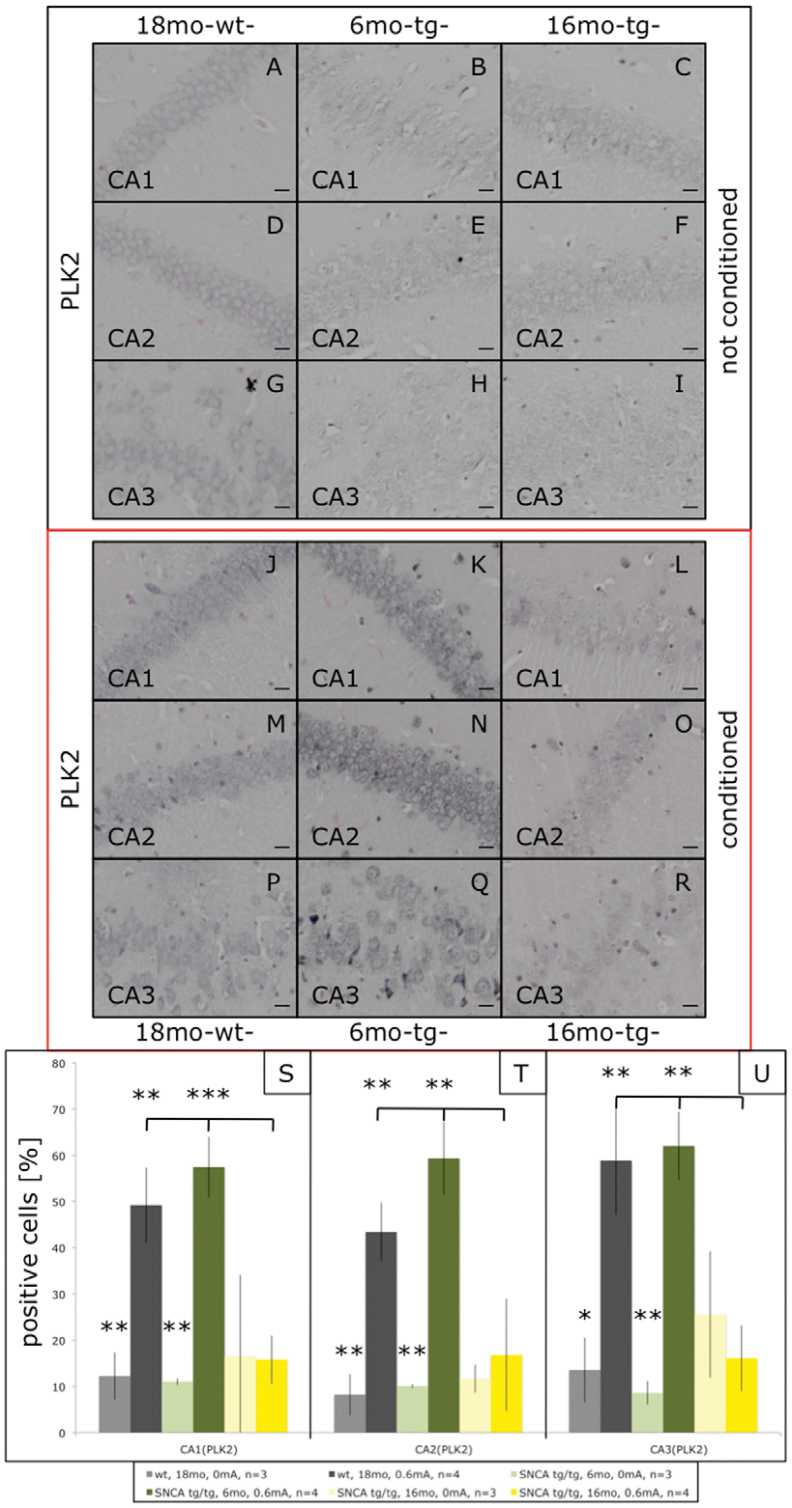
Plk2 immunostaining in the hippocampus of fear-conditioned (Thy1)-h[A30P]αSYN mice. Mice were processed as described above and hippocampal sections stained with an antibody against Plk2. Compared to non-shocked mice (A–I), FC induced a Plk2 up-regulation in the hippocampal regions CA1, CA2, and CA3 of old non-transgenic mice (J–P) and young (Thy1)-h[A30P]αSYN mice (K–Q). This FC dependent up-regulation of Plk2 was significant for the old wt group (grey bars in S (**p<0.0066), T (**p<0.0024), U (*p<0.0169)) and the young transgenic mice (green bars in S (**p<0.0018), T (**p<0.0051), U (**p<0.0055)). Plk2 up-regulation was not seen in the hippocampal CA1, CA2 and CA3 regions of old transgenic mice (L–R) and failed to show any significance (yellow bars in S–U). Size bars correspond to 20 µm.

In addition to the impairments in cued FC these mice show age-dependent impairments in context FC (Fig. 1), which involves the hippocampus [25]. Thus, we extended the study to the hippocampus. FC induced c-Fos significantly throughout the hippocampal formation even in old control mice (Fig. 4). C-Fos inductions were slightly reduced in the cognitively normal young transgenic mice, but strongly blunted throughout the hippocampal formation of old (Thy1)-h[A30P]αSYN mice (Fig. 4). Similarly, Plk2 was induced throughout the hippocampal formation upon FC even in old control mice (Fig. 5). Plk2 inductions were not reduced in young transgenic mice, in fact there might be a trend of increased Plk2 induction in CA1 and CA2 of young (Thy1)-h[A30P]αSYN mice (Fig. 5). In sharp contrast, Plk2 inductions throughout the hippocampal formation were abolished in old, FC-impaired (Thy1)-h[A30P]αSYN mice (Fig. 5). For biochemical confirmation, we prepared Western blots from crude hippocampal lysates. For c-Fos we could not detect obvious increases beyond the basal levels of this abundant protein. However, Plk2 did show enhanced signals upon FC of young but not old transgenic mice (Fig. S2), supporting the immunohistochemical data.

**Figure 6:**
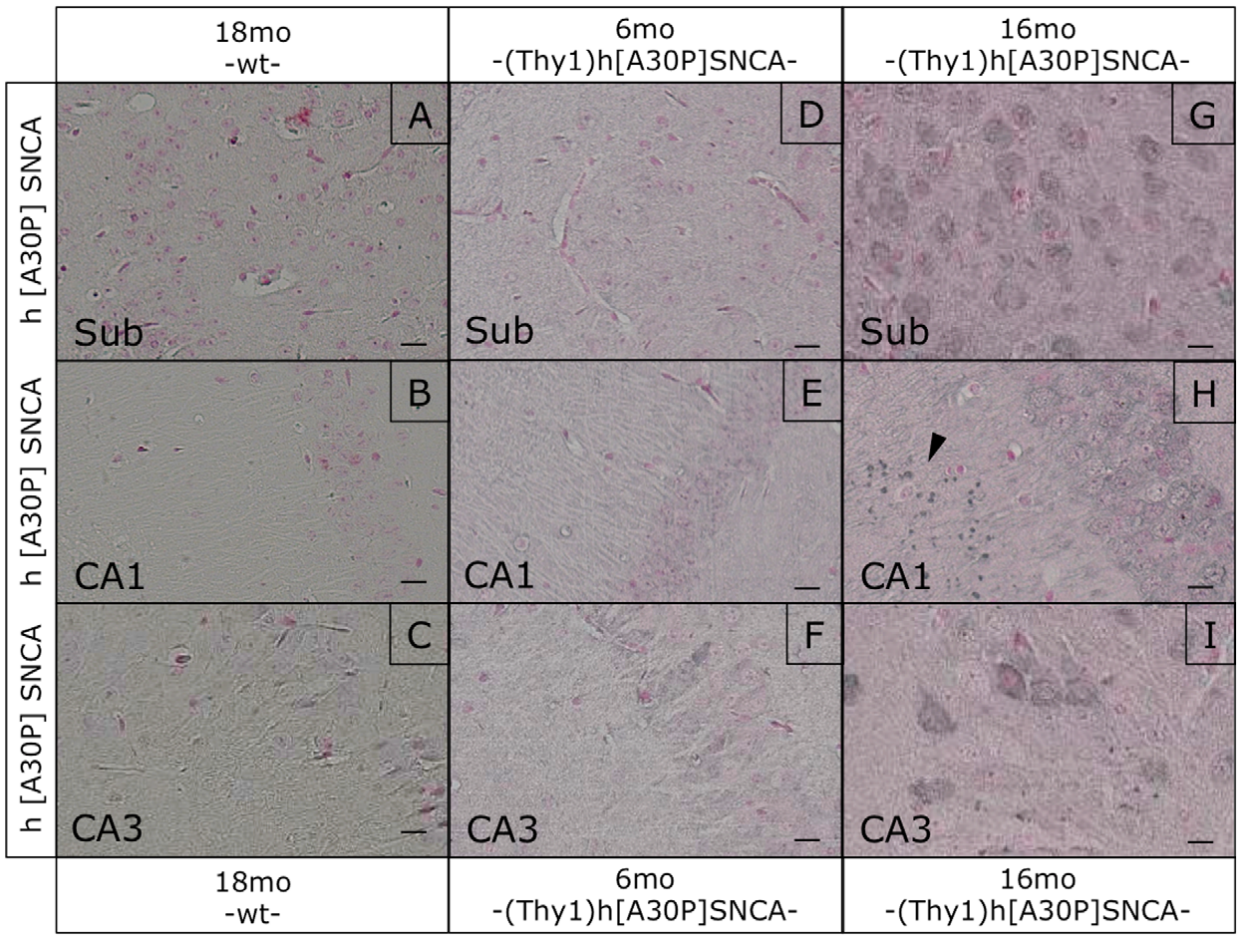
Transgenic human αSYN immunostaining in the hippocampus of (Thy1)-h[A30P]αSYN mice. Sections from old wt, young and old (Thy1)-h[A30P]αSYN transgenic mice were stained for human transgenic αSYN with the rat monoclonal antibody 15G7. As expected staining of tissue from old wt mice did not show any signal for human αSYN throughout the whole hippocampus (A–C). Tissue from young and old transgenic mice displayed a diffuse staining in the neuropil (D–I), and old transgenic mice showed some accumulation of human transgenic αSYN in the cytoplasm of neurons (G–I). Interestingly only the synaptic regions of the CA3 (I) and especially the CA1 (H) area of old transgenic mice were positive for transgenic αSYN positive dot-like structures (arrowhead in H). This staining pattern was not observed in similar areas of young transgenic mice (E,F). Size bars correspond to 20 µm.

**Figure 7:**
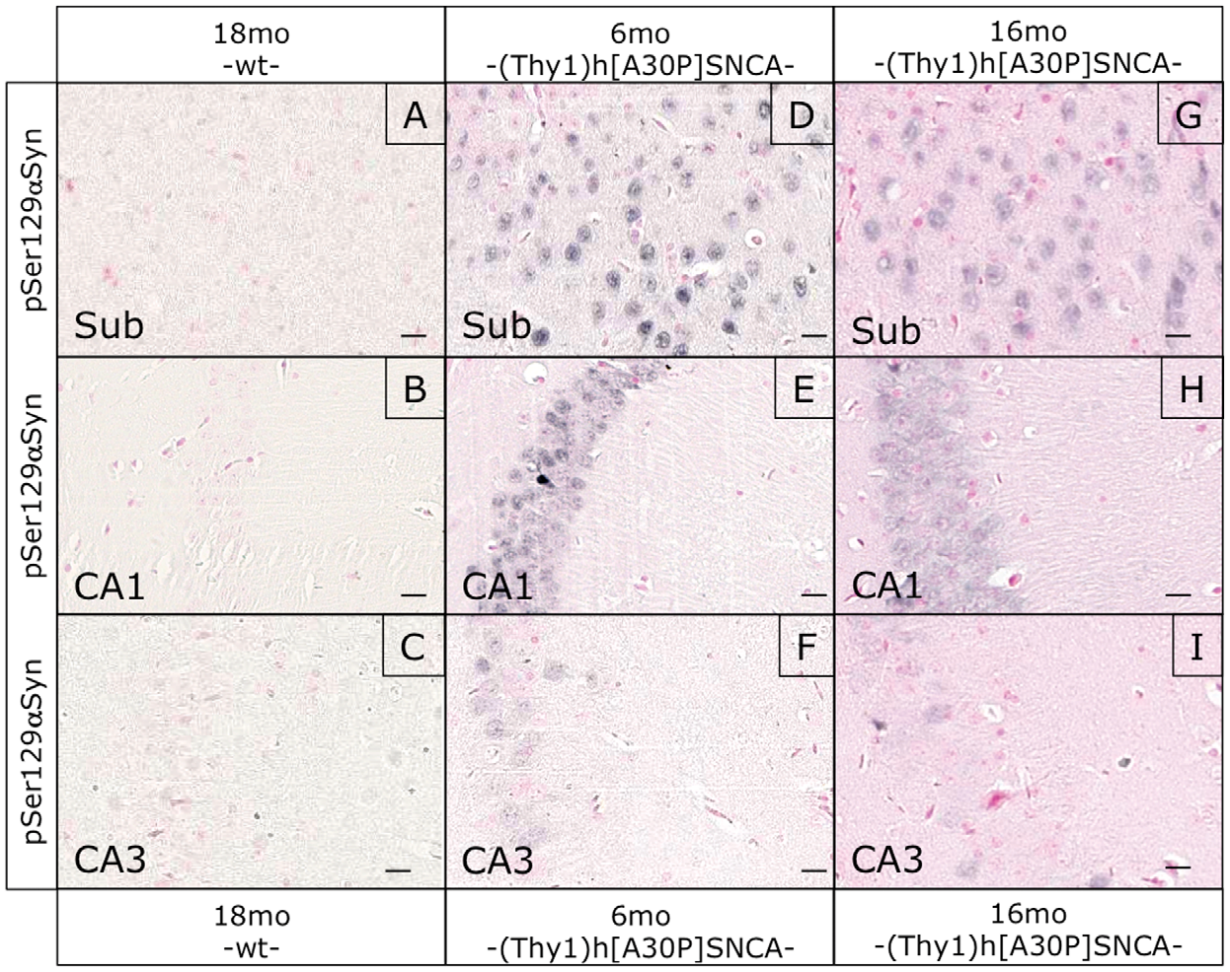
pSer129 staining in the hippocampus of young- and old (Thy1)-h[A30P]αSYN mice. Immunohistochemical staining with a pSer129 αSYN specific antibody displayed positive signals only in the cytoplasm of cells in the subiculum (Sub) (D,G), CA1 (E,H) and to a lesser extent the CA3 region (F,I) of young (D–F) and old (G–I) transgenic animals. As expected no area of the hippocampus even of old wt mice displayed any pSer129 αSYN positive signal (A–C). Size bars correspond to 20 µm.

**Figure 8:**
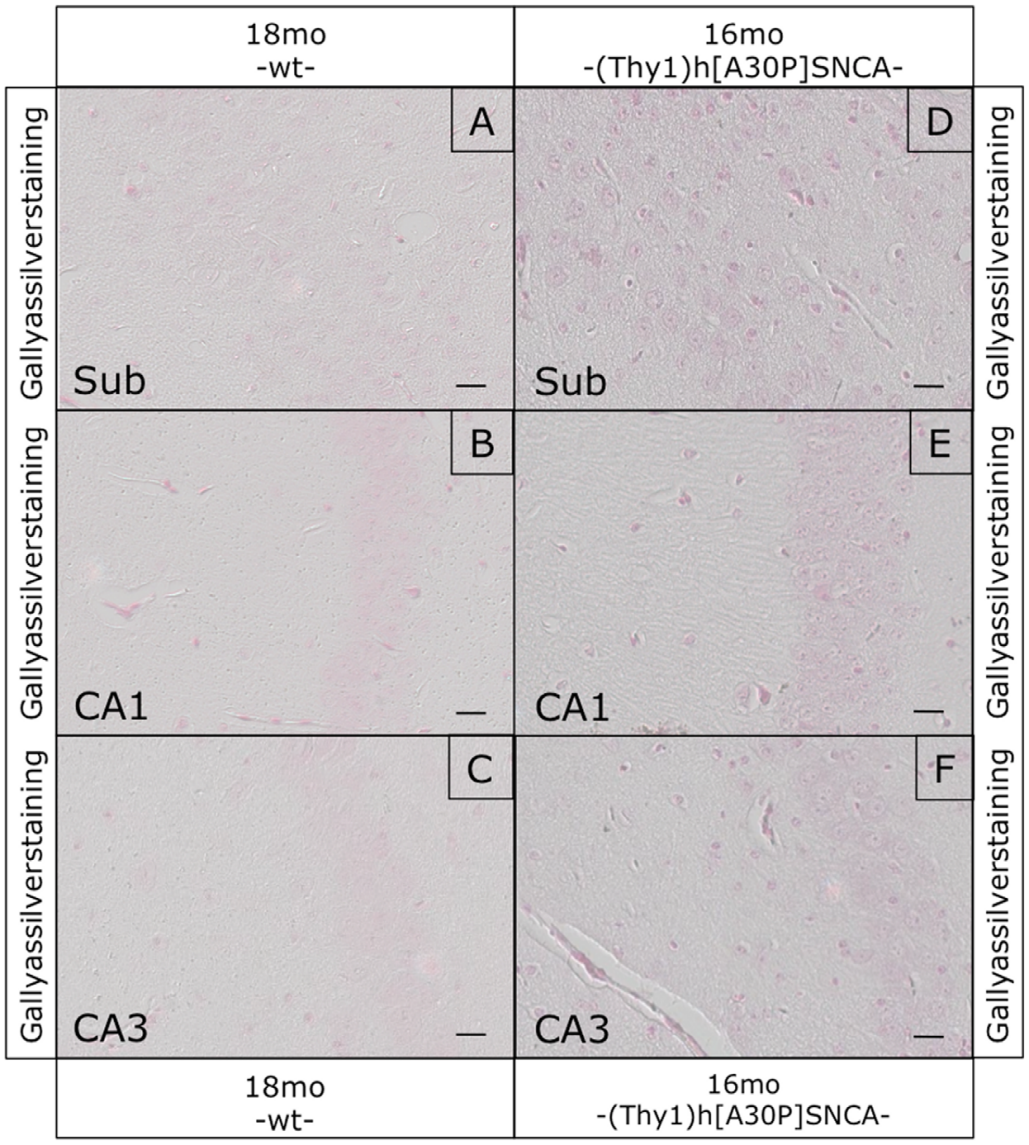
Gallyas silver staining of hippocampus from old wild type and old (Thy1)-h[A30P]αSYN mice. Brain sections of old transgenic animals (D–F) as well as sections from old wt control mice (A–C) were subjected to Gallyas silver staining. No silver signals could be observed. Size bars correspond to 20 µm.

Attempting to correlate these apparent hippocampal defects with α-synucleinopathy markers, we performed histological analyses of the hippocampal formation in these mice. Immunostaining with human transgene-specific antibody revealed an age-dependent accumulation of αSYN in synaptic profiles particularly in CA1, very similar to the dot-like αSYN profiles described recently [4], [5]. Such αSYN staining patterns were not observed in normally behaving young transgenic mice and completely absent in non-transgenic mice (Fig. 6). Interestingly, such profiles were not detected with anti-pSer129 (Fig. 7). Pre-synaptic accumulations of proteinase K-resistant αSYN in the hippocampus of human patients and (PrP)-h[A53T]αSYN mice were also negative for pSer129 in another study [5]. Here anti-pSer129 did not stain the dot-like profiles in the hippocampus, but only visualized nuclear-enriched staining patterns particularly in CA1 and subiculum, both in young and old (Thy1)-h[A30P]αSYN mice (Fig. 7), as reported before [16], [23]. Likewise, Gallyas silver staining did not reveal positive signals (Fig. 8) and “amyloid” α-synucleinopathy could also not be detected with thioflavin S staining in the hippocampal formation, even in old (Thy1)-h[A30P]αSYN mice (Fig. S3). Thus, these dot-like profiles might be synaptic accumulations of apparently “normal” αSYN.

## Discussion

These results demonstrate that the age-dependent cognitive decline of (Thy1)-h[A30P]αSYN transgenic mice is correlated with a parallel impairment in amygdala and hippocampus synaptic plasticity *in vivo*, as seen by immunohistological analysis of the immediate-early gene product c-Fos and the neuronal activity responsive kinase Plk2. These findings are consistent with previous *ex vivo* reports about affected synaptic plasticity electrophysiology in hippocampal slices from aged mice expressing transgenic h[A30P]αSYN under control of a mouse prion protein promoter [27] and in corticostriatal slices from different αSYN transgenic mice [28]. Very recently, exogenous addition of αSYN oligomer preparations was reported to impair long-term potentiation [29], [30]. It remains to be further investigated whether the impact of αSYN neuropathology on synaptic plasticity is due to effects on intra-neuronal signal transduction and/or via extra-cellular receptor modulation by secreted αSYN species.

Within the amygdala, impaired induction of the synaptic plasticity marker c-Fos was detected both in the BLA and the medial sector of the central amygdaloid nucleus (CEm). It is believed that synaptic plasticity not only occurs in the BLA but also in the CEm [31], which is consistent with our findings in wild-type mice. While proteinase K-resistant, serine-129 phosphorylated αSYN profiles looking like “Lewy neurites” were detected only in the CEm [11], [16]. Disturbed relay of the FC circuitry through CE may explain the observed impairments in both cued and contextual FC.

In addition to the finding of Plk2 induction by FC we measured a slight but significant increase of the Plk2 target pSer129 in young but not old transgenic mice. This raises the question of the relationship between αSYN phosphorylations and its kinases under physiological and pathological conditions. It is possible that induction of the nuclear associated Plk2 during long-term potentiation leads to a physiological serine-129 phosphorylation of αSYN in the nucleus. Very little is known about functions of αSYN in the nucleus [32], where it could potentially modulate epigenetics and transcription. Perhaps (excitotoxic) excessive phosphorylation of αSYN by Plk2 in the nucleus increases pSer129 shuttling to the cytosol, eventually ending up in cytosolic Lewy bodies. Alternatively, Plk2-mediated phosphorylation of αSYN may constitute a perfectly normal response for neurons involved in synaptic plasticity while the pathological pSer129 formation in the cytosol is a separate event mediated by distinct extra-nuclear kinases, such as Plk3 [23], G protein-coupled receptor kinases [33] and/or casein kinases [34]. It is essential to understand the exact mechanisms leading to pSer129 in the different neuronal compartments (nucleus, soma/cytosol. neurites and synapses) in health and disease to select and specifically exploit αSYN kinase candidates as potential drug targets [35].

Finally, it is even possible that elevated levels of “normal” αSYN interfere with synaptic plasticity, as seen directly here by examination of the hippocampus. Elevated levels of “normal” αSYN were shown to interfere with neurotransmitter release, although the A30P mutant used here did not seem to be effective in this regard [36]. Nevertheless, we do observe synaptic accumulations of apparently “normal” [A30P]αSYN in the hippocampus of old, cognitively impaired transgenic mice but not at younger ages when these mice are cognitively normal. Similar αSYN profiles were observed and correlated with impaired contextual FC in an independent αSYN transgenic mouse model [4].

In conclusion, the present study provides first direct *in vivo* evidence that pathological αSYN species or even excess of “normal” αSYN can impair synaptic plasticity in a learning paradigm, which might contribute to cognitive decline not only in transgenic mouse models, but also in demented α-synucleinopathy patients.

## Supporting Information

Figure S1 **pSer129 immunostaining in the amygdala of fear-conditioned (Thy1)-h[A30P]αSYN mice.** Mice were processed as above and amygdala sections stained with anti-pSer129. Compared to non-shocked mice, FC induced pSer129 signals in the BLA slightly but significantly (*p<0.04), which was not seen in old (Thy1)-h[A30P]αSYN mice. The staining pattern of pSer129 was mostly nuclear (arrows) with occasional neurons also showing diffuse cytosolic signals (arrowheads). Size bar corresponds to 200 µm.
(TIFF)

Figure S2 **Western blot analysis of the hippocampus from young- and old (Thy1)-h[A30P]αSYN mice.** For biochemical analysis, brains from the indicated mouse cohorts naïve (–) and fear-conditioned (+) were harvested and hippocampal tissue dissected. To obtain 50 µg hippocampal lysate (in RIPA: 1% NP-40, 0.5% deoxycholate, 150 mM NaCl, 50 mM Tris/Hcl (pH 7.5)+Cømplete protease inhibitor cocktail, Roche), 2 mice per condition were pooled. Samples were separated by denaturing 12.5% polyacrylamide gel electrophoresis and blotted onto polyvinylidene fluoride membranes (Immobilon, Millipore). Blots were probed with antibodies against human αSYN, c-Fos and Plk2, as indicated, and reprobed with mouse monoclonal anti-GAPDH as loading control. Peroxidase-conjugated secondary antibodies (Jackson ImmunoResearch) were used diluted 10∶000. Immunoblots were reacted with Immobilon Western chemiluminescent substrate (Millipore). The positions of Precision Plus protein standards (Dual Color, Bio-Rad) are indicated to the left.
(TIFF)

Figure S3 **ThS staining in the hippocampus of young- and old (Thy1)-h[A30P]αSYN mice.** To identify a possible amyloidosis induced by the overexpression of [A30P]αSYN in the hippocampus of young- and old transgenic mice, tissue isolated from those animals was stained with ThS. Neither young (A) nor old (B) transgenic mice displayed any ThS positive signals throughout the hippocampal formation. Size bars correspond to 200 µm.
(TIFF)
