# Supplementary figures and images for: Impaired c-Fos and Polo-Like Kinase 2 Induction in the Limbic System of Fear-conditioned α-Synuclein Transgenic Mice
Source: PLoS One. 2012 Nov 27;7(11):e50245. doi: 10.1371/journal.pone.0050245 (PMC3507699; doi:10.1371/journal.pone.0050245)

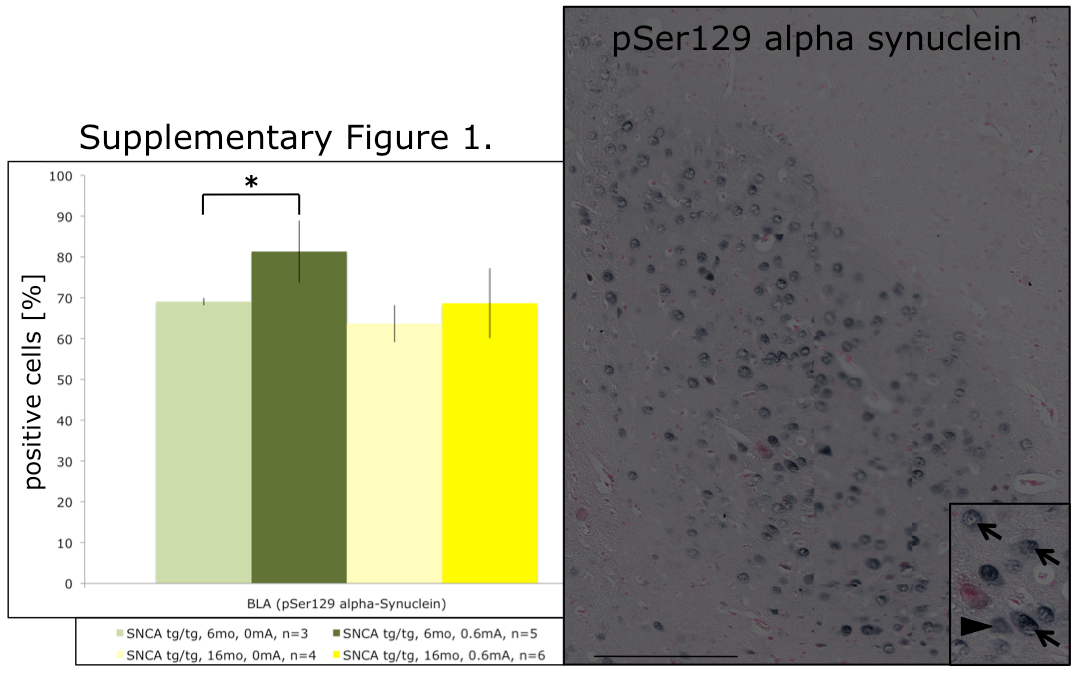

Supplement: Figure S1 — pSer129 immunostaining in the amygdala of fear-conditioned (Thy1)-h[A30P]αSYN mice. Mice were processed as above and amygdala sections stained with anti-pSer129. Compared to non-shocked mice, FC induced pSer129 signals in the BLA slightly but significantly (*p<0.04), which was not seen in old (Thy1)-h[A30P]αSYN mice. The staining pattern of pSer129 was mostly nuclear (arrows) with occasional neurons also showing diffuse cytosolic signals (arrowheads). Size bar corresponds to 200 µm. (TIFF) [file pone.0050245.s001.tiff]

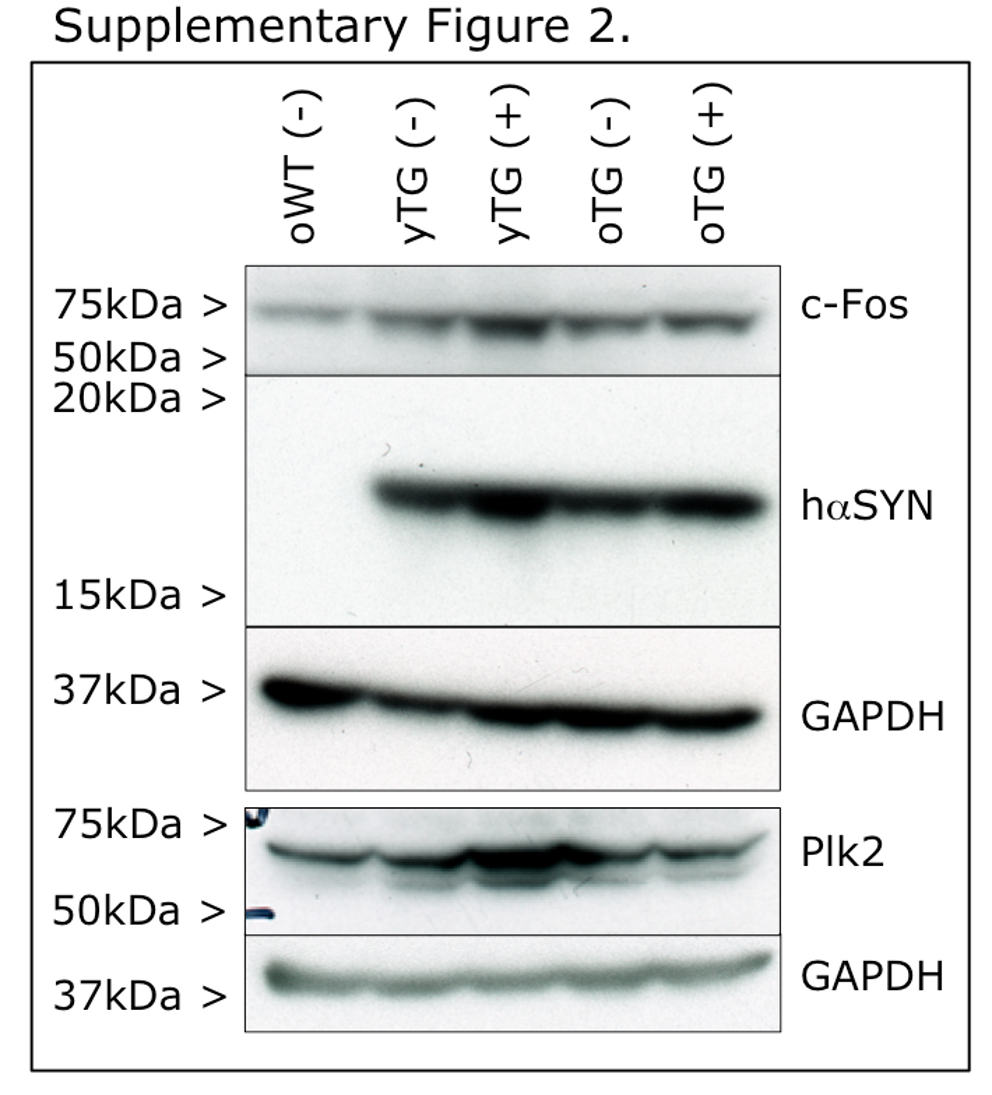

Supplement: Figure S2 — Western blot analysis of the hippocampus from young- and old (Thy1)-h[A30P]αSYN mice. For biochemical analysis, brains from the indicated mouse cohorts naïve (–) and fear-conditioned (+) were harvested and hippocampal tissue dissected. To obtain 50 µg hippocampal lysate (in RIPA: 1% NP-40, 0.5% deoxycholate, 150 mM NaCl, 50 mM Tris/Hcl (pH 7.5)+Cømplete protease inhibitor cocktail, Roche), 2 mice per condition were pooled. Samples were separated by denaturing 12.5% polyacrylamide gel electrophoresis and blotted onto polyvinylidene fluoride membranes (Immobilon, Millipore). Blots were probed with antibodies against human αSYN, c-Fos and Plk2, as indicated, and reprobed with mouse monoclonal anti-GAPDH as loading control. Peroxidase-conjugated secondary antibodies (Jackson ImmunoResearch) were used diluted 10∶000. Immunoblots were reacted with Immobilon Western chemiluminescent substrate (Millipore). The positions of Precision Plus protein standards (Dual Color, Bio-Rad) are indicated to the left. (TIFF) [file pone.0050245.s002.tiff]

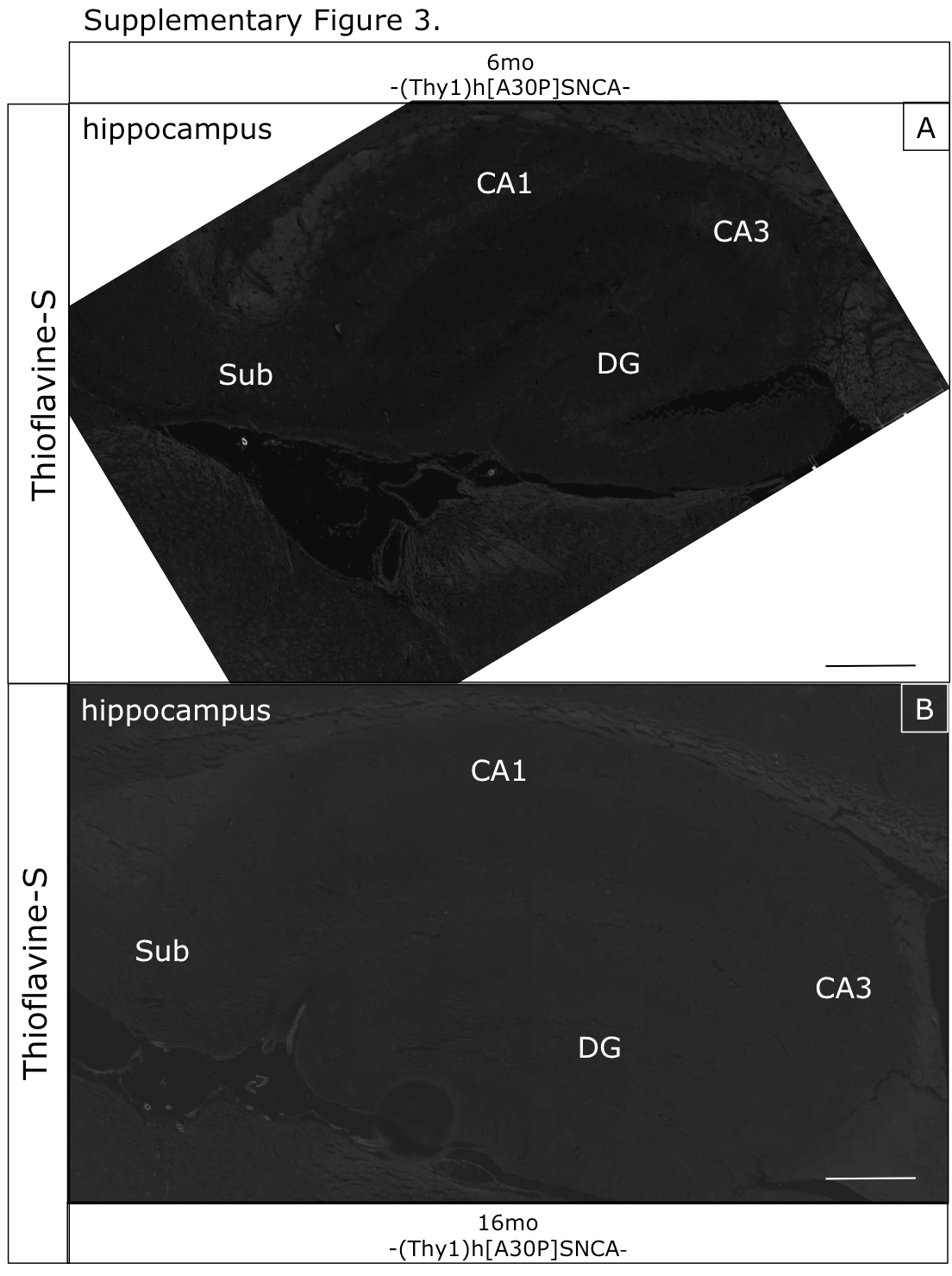

Supplement: Figure S3 — ThS staining in the hippocampus of young- and old (Thy1)-h[A30P]αSYN mice. To identify a possible amyloidosis induced by the overexpression of [A30P]αSYN in the hippocampus of young- and old transgenic mice, tissue isolated from those animals was stained with ThS. Neither young (A) nor old (B) transgenic mice displayed any ThS positive signals throughout the hippocampal formation. Size bars correspond to 200 µm. (TIFF) [file pone.0050245.s003.tiff]
